# Where to start? A two stage residual inclusion approach to estimating influence of the initial provider on health care utilization and costs for low back pain in the US

**DOI:** 10.1186/s12913-022-08092-1

**Published:** 2022-05-23

**Authors:** Kenneth J. Harwood, Jesse M. Pines, C. Holly A. Andrilla, Bianca K. Frogner

**Affiliations:** 1 College of Health and Education, Marymount University, Arlington, VA USA; 2 US Acute Care Solutions, Arlington, VA USA; 3 Department of Family Medicine, School of Medicine, University of Washington, Seattle, WA USA; 4 Center for Health Workforce Studies, Department of Family Medicine, School of Medicine, University of Washington, Seattle, WA USA

**Keywords:** Low back pain, Health care utilization, Opioids, Health care costs, Insurance claims, Conservative care

## Abstract

**Background:**

Diagnostic testing and treatment recommendations can vary when medical care is sought by individuals for low back pain (LBP), leading to variation in quality and costs of care. We examine how the first provider seen by an individual at initial diagnosis of LBP influences downstream utilization and costs.

**Methods:**

Using national private health insurance claims data, individuals age 18 or older were retrospectively assigned to cohorts based on the first provider seen at the index date of LBP diagnosis. Exclusion criteria included individuals with a diagnosis of LBP or any serious medical conditions or an opioid prescription recorded in the 6 months prior to the index date. Outcome measures included use of imaging, back surgery rates, hospitalization rates, emergency department visits, early- and long-term opioid use, and costs (out-of-pocket and total costs of care) twelve months post-index date. We used a two-stage residual inclusion (2SRI) estimation approach comparing copay for the initial provider visit and differential distance as the instrumental variable to reduce selection bias in the choice of first provider, controlling for demographics.

**Results:**

Among 3,799,593 individuals, cost and utilization varied considerably based on the first provider seen by the patient. Copay and differential distance provided similar results, with copay preserving a greater sample size. The frequency of early opioid prescription was significantly lower when care began with an acupuncturist or chiropractor, and highest for those who began with an emergency medicine physician or advanced practice registered nurse (APRN). Long-term opioid prescriptions were low across most providers except physical medicine and rehabilitation physicians and APRNs. The frequency and time to serious illness varied little across providers. Total cost of care was lowest when starting with a chiropractor ($5093) or primary care physician ($5660), and highest when starting with an orthopedist ($9434) or acupuncturist ($9205).

**Conclusion:**

The first provider seen by individuals with LBP was associated with large differences in health care utilization, opioid prescriptions, and cost while there were no differences in delays in diagnosis of serious illness.

**Supplementary Information:**

The online version contains supplementary material available at 10.1186/s12913-022-08092-1.

## Background

Low back pain (LBP) is a common, often self-limiting yet costly condition that significantly impacts the lives of individuals. Up to 80 % of the US population will have LBP at least once in a lifetime [1]. Nearly a quarter of individuals with LBP report physical function limitations [2]. Total annual costs for LBP was estimated at $100 to $200 billion in the US [3] with LBP health care costs growing at a pace greater than non-LBP expenditures [2].

LBP is a common reason for individuals to seek health care services in the US [4] For example, one study found that one in every 17 visits to a general medicine provider was for LBP [5]. For physical therapists and chiropractors, LBP is the most common diagnosis for individuals seeking care [6, 7]. Currently, there is growing interest in the effects of health care utilization and costs and the provider type that initiates care. For a majority of the US population, a physician typically initiates health care services [8]. However, other providers are increasingly acting as entry points into the health care system [9, 10].

A growing body of literature has found significant variation in LBP health care utilization and costs associated with the providers initiating care, suggesting that provider role and ordering in the care pathway may be important factors when considering methods to reduce costs. Frogner and colleagues [11] examined 2009–2013 private health insurance claims data from the Health Care Cost Institute (HCCI) in six Pacific Northwest states. The researchers found that adult, non-elderly individuals with LBP who were seen by a physical therapist (PT) first, as compared to individuals who saw a PT later or never, had an 89% lower probability of receiving an opioid prescription, 28% lower probability of having any advanced imaging services, and 15% lower probability of emergency department (ED) visits. There were also significantly lower outpatient, pharmacy, and out-of-pocket costs. Liu and colleagues [12] used 2008–2014 private health insurance claims data from MarketScan to compare cost and utilization differences for individuals with LBP who never saw a PT or saw a PT immediately (within 3 days from index date), early (4–14 days), delayed (15–28 days), or late (29–90 days). Among individuals who saw a PT, the authors found seeing a PT immediately had the lowest opioid medication use, ED use, pain medication, advanced imaging and non-LBP-related costs. Kazis and colleagues [13] used 2008–2013 private health insurance claims data from Optum to investigate the effects of initial provider seen on short- and long-term opioid use for individuals with LBP. The researchers found that individuals who saw a PT or chiropractor had lower odds of receiving short- and long-term opioids as compared to a primary care provider. In addition, physician specialty may affect the downstream costs and utilization. Fox and colleagues [14] found that mandatory physiatry consultation prior to LBP surgery decreased surgical rates by 25% and decreased overall cost of care.

In this study, we revisited the question of the influence of the first provider on downstream health care utilization and costs using a large national dataset. Where other studies used propensity score matching to control for selection bias, [12, 13] we applied a well-known econometric technique for causal inference called a two-stage residual inclusion (2SRI) estimation approach, which is an instrumental variable (IV) approach best suited for non-linear models while controlling for selection bias inherent in observational studies [15]. A related study used “differential distance”, defined as the difference in the distance between the patient and the provider seen versus the patient and a hypothetical alternative provider, as their instrument to control for selection bias in their 2SRI model and predict downstream health care utilization and costs for LBP care on choice of first provider [11]. In this study, we examine whether copay for the initial visit to a provider is a suitable alternative instrument to predict the initial choice of provider, and conduct a sensitivity analysis of results using differential distance as the instrument. Copay is an instrument that has been previously used in the Pharmacoeconomics literature [16–18]. Costs related to care are notoriously opaque despite the growing availability of price transparency tools. Individuals, however, have information on the copay for generalists and specialists, which may influence where they start with care. Once at a provider’s office, individuals trust their providers to guide them in their care [19]. Patients have limited ability to predict subsequent out-of-pocket costs for care, especially when faced with multiple treatments such as prescriptions, imaging services or ongoing services such as PT. While both patients and providers have expressed the desire for transparent cost/price information when making treatment decisions, minimal, if any, causal evidence exists on whether being aware of costs affects treatment decisions and thus downstream costs of care [20–22].

The specific aims of our study were to determine the extent to which the first provider seen for LBP impacts health care costs and utilization, including short- and long-term opioid use, imaging, hospitalizations, out-of-pocket costs, and total costs, using a large national health insurance claims database. In the 2SRI approach, we use copay for the initial provider visit as the primary instrument to control for selection bias to the first provider. While randomized control trials are the gold standard for comparing interventions, this study provides a large-scale, real-world look at the complexity of and variation in LBP care that may be influenced by the first health care provider seen.

## Methods

### Sample and study design

We reviewed all eligible insurance claims for LBP using the 2015–2016 Health Care Cost Institute (HCCI) database that includes health insurance claims data for approximately 50 million insured individuals per year in the US, across four private health insurance companies (Aetna, Humana, United Healthcare, and Kaiser Permanente) including claims covered by a Medicare Advantage plan. The data that support the findings of this study are available from HCCI (https://healthcostinstitute.org), but restrictions apply to the availability of these data, which were used under license for a fee to conduct the current study, and so are not publicly available. HCCI is an independent, non-profit organization that licenses access to insurance claims data via secure enclave. We merged county-level data from the 2015 Area Health Resource File (AHRF) and distance information from the National Bureau of Economic Research (NBER) [23, 24].

We restricted the sample to include individuals 18 years or older who lived and received services within the 50 states and the District of Columbia. Individuals were excluded if they had a diagnosis of LBP, serious illness associated with non-musculoskeletal based LBP (S1 Table A), or an opioid prescription as defined by National Drug Codes identified by the Centers for Disease Control and Prevention [25] 6 months prior to the index date (date of diagnosis of low back pain), which we refer to as a “clean period”. We further restricted the sample to those individuals who had continuous insurance enrollment for one-year after the index date. LBP was defined using ICD9/10-CM codes from the literature that were frequently used to designate nonspecific LBP diagnoses. (S1 Table B) [26, 27].

We defined cohorts based on the first provider seen on the index date. Provider categories were included in the study based on the most frequent health care providers seen first for an episode of musculoskeletal LBP in the database. The provider categories examined were: 1) acupuncturist (Acu), 2) advanced practice registered nurse (APRN), 3) chiropractor (Chiro), 4) emergency medicine (EM), 5) orthopedic specialist (Ortho), 6) physical medicine and rehabilitation (PM&R), 7) physical therapist (PT), and 8) primary care physician (PCP) including family medicine, internal medicine, and osteopathic medicine. All other provider types not specified above were collapsed into a category of “Other”. The six most common other provider types that made up about 75% of this category included radiology (23%), anesthesiology (17%), unknown provider type (15%), other non-physician provider (6%), and neurological surgeon (5%). We assumed that these providers were not common choices for an individual with a new diagnosis of LBP, but we kept this other category in the analysis to maximize sample size. For PT, we used the Current Procedural Terminology (CPT) code 97001, which is an evaluation code for new PT visits and excluded individuals with the follow-up examination CPT code (97002).

In some cases, individuals saw more than one provider on the index date. We excluded these individuals to ensure a uniform analysis of treatment decisions associated with the first provider. Of the 525,663 excluded, approximately 25% saw a PCP, 20% saw an EM, and 40% saw an “Other” type of provider; as such, the activities of these groups may be slightly underrepresented. Our final sample size was 3,799,593. Our study was approved by The George Washington University Institutional Review Boards (IRB #011814). All experiment protocol for involving humans was in accordance to guidelines of national/international/institutional or Declaration of Helsinki in the manuscript. A waiver for consent was given by The George Washington University IRB. Our research team had a signed data agreement with HCCI that is re-examined annually to access the proposed dataset. HCCI was the holder of the identifiers, and prohibited the release of the identifiers to the study team.

### Measures

As our dependent variables, we included several utilization and cost measures within the one-year post-index date. We included a set of binary measures related to opioid prescriptions. To allow for comparison across studies, we used the definition by Kazis and colleagues for early- and long-term opioid prescriptions [13]. Receiving an early opioid prescription was defined as a filled prescription within 30 days or less of the index date. Receiving a long-term opioid prescription was defined as a filled prescription within 60 days or less and either 1) received 120 days or more of pills supplied in the one-year post-index date or 2) received 90 or more days of pills supplied and had 10 or more refills in that 1 year. We created binary measures for a) diagnostic imaging services, which was defined as whether an individual had magnetic resonance imaging (MRI) or a computed tomography (CT) scan, b) radiography, c) surgery related to LBP (Table A in S1), d) ED visit, and e) inpatient hospitalization. A binary measure was created for any serious illness related to LBP (see Table A in S1 for ICD-9 or − 10 codes) to address concerns about any delays in diagnoses associated with provider types.

We used two measures of costs. First, we defined total health care costs as the net paid amount to the provider after all deductions and calculations over the course of the one-year post-index date. Second, we defined total out-of-pocket costs, which are a subset of total health care costs, to include deductibles, coinsurance, and copays for all visits over the course of the year. Negative or missing values were coded as zero.

Control variables included whether an individual identified at the index date as female or male, age categories (with age 18 to 34 as the reference category and age 75 and older collapsed into one category), whether an individual was in a Medicare Advantage plan, plan type categorized by flexibility of provider referrals (preferred provider organization and exclusive provider organization as the combined reference category; health maintenance organization; point of service; and “other” including private fee-for-service, independent, life insurance, and unknown), whether the individual was in a high deductible plan or not, and an Elixhauser Index value based on all other diagnoses identified on the index date (and accounting for changes in coding from ICD-9 to ICD-10-CM) [28].

Using data from the AHRF, we categorized the county in which an individual lived based by Urban Influence Code (UIC), which accounts for population density and urban influence, collapsed into three categories: metropolitan (reference), micropolitan and noncore (rural) [23, 29]. To account for socioeconomic factors influencing the utilization of health care by individuals, we included county typology codes defined by the Economic Research Service (ERS) including whether the county population had low levels of educational attainment (defined as 20 % or more of the working-age population lacking a high school diploma or equivalent), low employment levels (defined as less than 65% of working age population employed), and whether the county population had persistent poverty levels. We included the percent of the county that was uninsured. We also controlled for whether a patient lived in a state with limits or provisions on physical therapy services.

### Instrumental variables estimation

Observational studies require statistical techniques to control for confounding, or selection bias, into an intervention. To control for the selection bias associated with an individual’s choice of the first provider seen, which is the variable of interest in our study, we used an econometric technique called instrumental variable (IV) estimation that is well-known for causal inference in health services research and epidemiological studies [30, 31]. We specifically used the two-stage residual inclusion (2SRI) estimation approach of the IV estimation, which is recommended for use in non-linear modeling to create consistent estimators [15]. An IV estimation requires a variable (the “instrument”) that strongly predicts the intervention that an individual receives (i.e., the first provider seen at the index date), but is not directly associated with the outcome measures. We used the copay associated with the index date as our “instrument”. For a sensitivity analysis, we also tested differential distance, an instrument used in a previous study, [11] defined as: 1) the distance between an individual and the first provider of choice, and 2) the distance between an individual and the closest alternative provider (see S2 for further discussion about measures and S2 Table A and Table B for first stage results).

Both instruments had a large Wald statistic. Given that the Wald statistic is asymptotically equivalent to a likelihood ratio test statistic, a generalized version of the F-statistic, we have evidence that our instrument satisfies the F-test threshold being above 10, indicating a strong instrument [32, 33]. Given that the sample size was large for this study, achieving this threshold was not surprising.

### Statistical analysis

We report descriptive statistics for bivariate analyses. In the first stage of the 2SRI, we predict the first provider type seen (PT as reference group) as a function of the instrument (e.g., copay) and control variables described in the previous section using a multinominal logistic regression model, which is used to estimate categorical variables with no logical ordering (see S2 Tables A & B). In the second stage, we used probit models to predict the probability for each of our health care utilization measures (e.g., opioid prescription, imaging service, ED visit, hospitalization, surgery, and serious illness) and generalized linear models assuming gamma distribution and using a log link to estimate total and out-of-pocket costs as a function of first provider seen, control variables from the first stage, and the raw residuals from the first stage. We conducted two additional sensitivity analyses: 1) using deviance residuals in place of raw residuals, which produced nearly identical results, and 2) adding state dummies produced similar consistent parameter estimates but we did not include them in the final model at risk of overfitting. While we kept the “Other” category of providers in our models to preserve sample size and further reduce selection bias, we report predicted probabilities for each of the binary outcome measures and the predicted costs for individuals seen by each of the specific provider types; results for the Other group are available upon request. Robust standard errors were used for all models.

## Results

### Sociodemographic statistics

In our sample of 3,799,593 individuals, 25.2% saw PCP first, followed by Chiro (24.8%), Ortho (5.1%), PM&R (4.2%), PT (2.9%), EM (2.4%), APRN (1.6%), Acu (1.0%) and 32.8% saw a mix of “other” providers (Table 1). The total sample included a greater percentage of females (56.0%) versus males (44.0%), older individuals in the 55 to 64 age group (21.5%), individuals living in a metropolitan area (86.0%) versus non-metropolitan area, and individuals in point of service health insurance plans (54.8%) versus other plan types. There were differences in the demographic characteristics of patients seen by various provider types as shown in Table 1. For example, a larger percentage of patients seen by chiropractors (22.9%) and acupuncturists (23.0%) were in the 18–34 age group, and a larger percentage of patients seen by APRNs were from micropolitan (11.1%) and non-core (11.5%) areas than for other provider types.

**Table 1:** Sociodemographic characteristics by first provider seen for low back pain

	Total		PT		Chiro		ACU		APRN		PCP		PM&R		Ortho		EM		All Other	
Sample Size	3,799,593		109,480		942,925		37,522		60,734		957,619		160,537		192,144		91,979		1,246,653	
	Mean (%)	SD (%)	Mean (%)	SD (%)	Mean (%)	SD (%)	Mean (%)	SD (%)	Mean (%)	SD (%)	Mean (%)	SD (%)	Mean (%)	SD (%)	Mean (%)	SD (%)	Mean (%)	SD (%)	Mean (%)	SD (%)
Female	56.0	49.6	60.4	48.9	53.4	49.9	63.3	48.2	58.5	49.3	54.3	49.8	58.4	49.3	56.1	49.6	53.9	49.8	58.3	49.3
Age 18–34	16.4	37.1	18.4	38.7	22.9	42.0	23.0	42.1	18.6	38.9	14.7	35.4	9.8	29.8	11.9	32.4	27.4	44.6	13.2	33.8
Age 35–44	16.4	37.1	17.3	37.8	20.2	40.2	30.2	45.9	18.4	38.7	16.4	37.0	13.3	33.9	12.8	33.4	21.7	41.2	13.6	34.3
Age 45–54	20.5	40.3	19.9	39.9	21.1	40.8	25.3	43.5	22.3	41.7	21.2	40.9	20.3	40.2	19.3	39.5	20.7	40.5	19.3	39.5
Age 55–64	21.5	41.1	20.7	40.5	18.2	38.6	15.5	36.2	21.8	41.3	22.8	42.0	24.8	43.2	23.8	42.6	15.8	36.5	23.0	42.1
Age 65–74	14.9	35.6	14.2	34.9	11.3	31.7	4.6	20.9	10.9	31.1	14.9	35.6	19.2	39.4	19.1	39.3	8.6	28.0	17.4	37.9
Age 75+	10.2	30.3	9.5	29.3	6.2	24.1	1.4	12.0	8.0	27.2	10.0	30.0	12.6	33.2	13.0	33.6	5.9	23.5	13.5	34.2
Elixhauser Index (#)	0.04	0.22	0.02	0.14	0.02	0.14	0.01	0.10	0.04	0.21	0.05	0.23	0.04	0.21	0.03	0.16	0.03	0.18	0.07	0.26
Medicare Advantage	23.0	42.1	15.6	36.3	12.7	33.3	1.9	13.6	16.9	37.5	24.4	42.9	33.3	47.1	27.4	44.6	14.7	35.4	30.0	45.8
PPO/EPO	27.9	44.9	26.4	44.1	23.7	42.5	16.4	37.0	21.0	40.7	27.5	44.7	31.0	46.3	29.7	45.7	21.8	41.3	32.1	46.7
POS	54.8	49.8	60.8	48.8	67.6	46.8	80.7	39.5	64.5	47.9	52.8	49.9	46.2	49.9	51.0	50.0	58.9	49.2	46.3	49.9
HMO	14.0	34.7	9.0	28.6	6.1	24.0	2.4	15.4	11.1	31.4	16.8	37.3	19.6	39.7	15.5	36.2	17.6	38.1	17.6	38.1
Other	3.2	17.7	3.8	19.1	2.6	15.8	0.6	7.5	3.5	18.3	2.9	16.9	3.2	17.6	3.8	19.0	1.6	12.5	4.0	19.6
Not in High Deduct Plan	72.4	44.7	69.6	46.0	67.2	47.0	70.2	45.7	68.3	46.5	73.7	44.0	75.6	43.0	73.1	44.3	71.9	44.9	75.4	43.0
High Deductible Plan	22.3	41.6	24.1	42.7	28.6	45.2	28.3	45.0	24.3	42.9	21.2	40.9	18.9	39.1	21.2	40.8	25.5	43.6	18.3	38.7
Unknown High Deductible	5.3	22.3	6.4	24.4	4.2	20.1	1.5	12.1	7.3	26.1	5.1	21.9	5.5	22.9	5.7	23.2	2.5	15.7	6.2	24.1
Metropolitan (UIC)	86.0	34.7	91.8	27.5	84.7	36.0	98.0	14.1	77.5	41.8	85.4	35.3	90.3	29.6	89.7	30.3	90.7	29.1	85.4	35.3
Micropolitan (UIC)	6.5	24.7	3.8	19.1	7.7	26.6	0.7	8.2	11.1	31.4	6.6	24.9	4.7	21.2	4.8	21.4	4.4	20.4	6.4	24.4
Noncore (UIC)	7.5	26.4	4.4	20.6	7.6	26.5	1.3	11.5	11.5	31.9	8.0	27.1	5.0	21.8	5.4	22.7	5.0	21.8	8.2	27.4
County: Low Education	8.4	27.8	5.7	23.1	7.3	26.0	10.2	30.3	8.8	28.3	9.8	29.8	5.6	23.0	9.4	29.2	8.6	28.0	8.6	28.0
County: Low Employ	11.0	31.3	6.6	24.9	8.9	28.5	2.0	14.1	15.7	36.4	12.7	33.3	9.1	28.8	9.6	29.5	9.0	28.6	12.3	32.8
County: High Poverty	11.8	32.3	9.5	29.3	9.4	29.2	4.5	20.8	16.2	36.8	13.5	34.2	10.3	30.4	11.0	31.3	10.6	30.7	12.9	33.5
County Uninsured	11.0	5.0	9.5	4.4	10.3	4.9	8.6	4.1	10.8	4.9	11.4	5.0	10.8	4.6	11.6	5.0	11.2	4.9	11.5	5.0

### Comparison of instrument variables

We found the parameter estimates were nearly identical with either copay or differential distance as the instrument, although the standard errors were slightly narrower at the fifth or sixth decimal place using copay over differential distance. Choosing copay preserves more of the sample over differential distance given limitations in defining this instrument. The tradeoff was the loss of information from the nearly three-quarters of a million observations missing distance information. In the following sections, we present 2SRI results using copay as the instrument (see S2 Figs. A and B and Tables C and D for select comparative results using differential distance).

### Health care utilization and costs

**Fig. 1:**
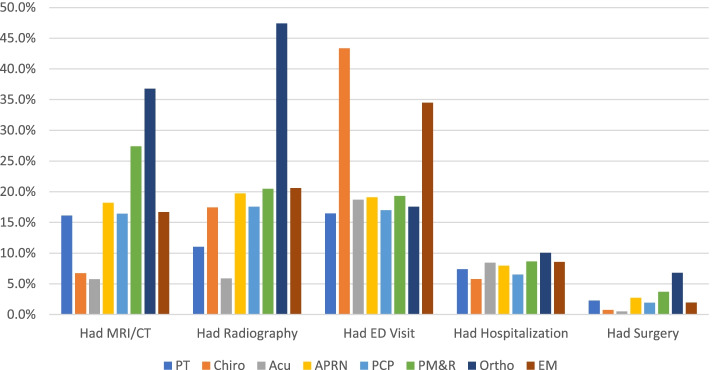
Health Care Utilization Adjusted Rates by First Provider Seen for Low Back Pain. PT = physical therapist; Chiro = chiropractor; Acu = acupuncturist; APRN = advanced practice registered nurse; PCP = primary care physician; PM&R = physical medicine and rehabilitation physician; Ortho = orthopedist; EM = emergency medicine physician “Other” category results available upon request. Margins refer to the marginal effect (dy/dx) at the means reported from the second stage (probit) of a two-stage residual inclusion instrumental variable approach. Models control for gender, age, co-morbidity, insurance status, rurality, county-level economic measures, and state limitations/provisions to physical therapy access; SE = standard error (robust)

Results from the 2SRI demonstrated considerable variation in health care utilization after accounting for selection for the first provider seen by individuals with LBP (Fig. 1). Table 2 includes the marginal effects from the second stage of the 2SRI model, with the corresponding standard error, that provide the predicted change in individuals with LBP utilizing selected health care services and experiencing serious illness within the 12-months after the first provider seen relative to the choice of initial provider. The results are summarized below.

**Table 2:** Marginal effects of patient utilizing health care services and experiencing serious illnesses in 12-months after first provider seen for low back pain

	Early Opioid Rx		Long Opioid Rx		Had MRI/CT		Had Radiography		Had ED Visit		Had Hospitalization		Had Surgery		Had Serious Illness	
	Margin	SE	Margin	SE	Margin	SE	Margin	SE	Margin	SE	Margin	SE	Margin	SE	Margin	SE
PT	3.2	0.058	1.4	0.040	16.1	0.112	11.0	0.096	16.5	0.114	7.4	0.081	2.3	0.046	22.9	0.125
Chiro	1.7	0.015	0.6	0.009	6.7	0.027	17.4	0.040	43.3	0.053	5.8	0.026	0.7	0.009	19.2	0.042
Acu	1.2	0.069	0.4	0.045	5.7	0.127	5.9	0.125	18.7	0.209	8.4	0.166	0.5	0.039	22.1	0.225
APRN	11.2	0.127	5.3	0.090	18.2	0.157	19.7	0.162	19.1	0.158	8.0	0.112	2.7	0.066	21.8	0.166
PCP	9.9	0.030	3.6	0.018	16.4	0.038	17.6	0.039	17.0	0.038	6.5	0.025	1.9	0.014	19.9	0.040
PM&R	11.1	0.073	6.3	0.055	27.4	0.111	20.5	0.101	19.3	0.096	8.7	0.067	3.7	0.047	24.6	0.103
Ortho	8.0	0.060	2.5	0.034	36.8	0.110	47.4	0.115	17.6	0.086	10.0	0.066	6.8	0.057	25.9	0.097
EM	12.6	0.109	1.9	0.048	16.7	0.126	20.6	0.134	34.5	0.154	8.6	0.096	2.0	0.048	21.7	0.140

Margins refer to the marginal effect (dy/dx) at the means reported from the second stage (probit) of a two-stage residual inclusion instrumental variable approach where the numerator (dy) is the change in outcomes as specified in the column headings and the denominator (dx) is the initial choice of provider. Models control for gender, age, co-morbidity, insurance status, rurality, county-level economic measures, and state limitations/provisions to physical therapy access. *SE* standard error (robust), *PT* physical therapist, *Chiro* chiropractor, *ACU* Acupuncturist, *APRN* advanced practice registered nurse, *PCP* primary care physicians, *PM&R* physical medicine and rehabilitation physician, *Ortho* orthopedist, *EM* emergency medicine physician. “Other” category results available upon request

#### Radiography and MRI/CT

The use of radiography and MRI/CT varied widely depending upon the first provider seen (Fig. 1, Table 2). Individuals that first saw Ortho (47.4%) had highest use of radiography; Acu (5.9%) and PT (11.0%) had the lowest rates of radiography use. Individuals who first saw Ortho (36.8%), had highest utilization of MRI/CT; whereas individuals that first saw Acu (5.7%) and Chiro (6.7%) were less likely to have MRI/CT.

#### Hospitalization, ED visits, Back surgery, serious illness

Hospitalization rates had relatively small variation among the providers with the overall rate being relatively low (7.4%) (Fig. 1, Table 2). Individuals that first saw Ortho (10.0%) were most likely to have had a hospitalization whereas individuals that first saw Chiro (5.8%) and PCP (6.5%) were least likely to be hospitalized. Individuals that first saw Ortho were more likely to have back surgery (6.8%) whereas individuals that saw Acu (0.5%) and Chiro (0.7%) were least likely to have had back surgery. ED visits during the follow-up period showed wide variation in frequency as individuals who saw a Chiro first had the highest probability of an ED visit (43.3%) followed by EM (34.5%), whereas the least likely was PT (16.5%) and PCP (17.0%).

The frequency of serious illness diagnoses that were associated with LBP symptoms (red flags and common non-musculoskeletal diagnoses that refer to LBP – Table A in S1) during the one-year follow-up was 22% (SD: 42%). The frequency did not vary significantly across providers (19–25%). The time to serious illness diagnosis (number of days from index date to diagnosis date) was 125 days (SD: 98 days). Similarly, the time to serious illness diagnosis was not significantly different among providers (104–144 days with SE: 95–98 days).

#### Opioids

**Fig. 2:**
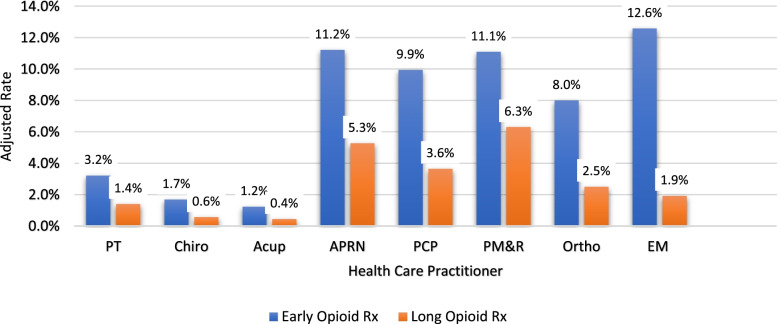
Early and Long Opioid Prescription (Adjusted Rates) by First Provider Seen for Low Back Pain. Margins refer to the marginal effect (dy/dx) at the means reported from the second stage (probit) of a two-stage residual inclusion instrumental variable approach where the numerator (dy) is the change in early opioid or long opioid prescriptions and the denominator (dx) is the initial choice of provider. Models control for gender, age, co-morbidity, insurance status, rurality, county-level economic measures, and state limitations/provisions to physical therapy access; SE = standard error (robust); PT = physical therapist; Chiro = chiropractor; ACU = Acupuncturist; APRN = advanced practice registered nurse; PCP = primary care physicians; PM&R = physical medicine and rehabilitation physician; Ortho = orthopedist; EM = emergency medicine physician; “Other” category results available upon request

The frequency of early opioid prescription was significantly lower for individuals who first saw Acu (1.2%) and Chiro (1.7%). Individuals that saw EM (12.6%) and APRN (11.2%,) had relatively high frequency of early opioid prescription (Fig. 2). Overall, long-term opioid prescriptions were lower for all providers compared to early opioid prescriptions. Individuals that first saw PMR (6.3%) and APRN (5.3%) had the highest rates of receiving a long-term opioid prescription, while individuals being seen first by Acu (0.4%) and Chiro (0.6%) were least likely to receive a long opioid prescription.

#### Rank order of providers for each health care outcome variable

In order to summarize the results of the health care utilization of the first providers seen by individuals with LBP, we rank-ordered the highest to lowest users for each health care utilization variable though noting that the difference between margin rates was minimal in some cases (Table 3). Generally, individuals who first started with a Chiro, Acu, or PT ranked lowest in health care utilization across most measures of interest.

**Table 3:** Health care category ranking by first provider seen (highest use = 1, lowest use = 8)

	PT	Chiro	Acu	APRN	PCP	PM&R	Ortho	EM
Early Opioid Rx	6	7	8	2	4	3	5	1
Long Opioid Rx	6	7	8	2	3	1	4	5
MRI/CT	6	7	8	3	5	2	1	4
Any Radiography	7	6	8	4	5	3	1	2
Had ED Visit	8	1	5	4	7	3	6	2
Hospitalization	6	8	4	5	7	2	1	3
Had Surgery	4	7	8	3	6	2	1	5
Had Serious Illness	3	8	4	5	7	2	1	6

*Note*: “Other” category results available upon request

#### Health care costs

**Fig. 3:**
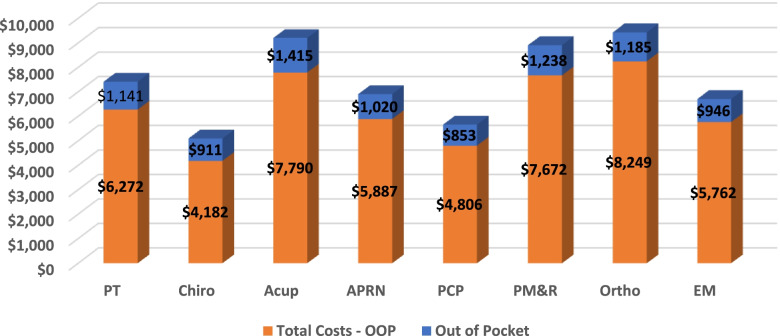
Adjusted Health Care Costs: Total and Out of Pocket (OOP) Costs by First Provider Seen for Low Back Pain. PT = physical therapist; Chiro = chiropractor; Acu = acupuncturist; APRN = advanced practice registered nurse; PCP = primary care physician; PM&R = physical medicine and rehabilitation physician; Ortho = orthopedist; EM = emergency medicine physician. Other category results available by request

Total cost of care was lowest for individuals (Fig. 3) who first saw Chiro ($5093) and PCPs ($5660) and highest for individuals who saw Ortho ($9434) first. Out-of-pocket costs were the least for individuals that saw a PCP ($853) and Chiro ($911) first and highest for those individuals that saw Acu ($1415), and PM&R ($1238) first.

### Limitations

While claims data are among the largest data available to examine LBP treatment, the study has several limitations related to the use of this data. The use of an IV approach helps to eliminate selection bias due to omitted variables (or measures not available), yet there are a number of variables not captured in claims data that would provide a better understanding of the patient experience. One example is identifying who actually provided the services. For example, one recent study in Massachusetts found that approximately 25 of continuing evaluation and management codes for APRN office visits were billed “incident to” a physician between 2015 and 2017 [34]. Thus, our results may not be a full representation of care by APRNs. Another challenge is the lack of safety and functional outcome measures. The one set of safety measures available was frequency and timing to serious illness. Frequency of serious illness diagnoses were similar across providers and timing was approximately 125 days post initial LBP diagnosis across most providers. Additional data collection is needed to obtain information on functional recovery and return to work.

Studying LBP also poses challenges. LBP is a condition that may recur over one’s lifespan so determining the exact onset of LBP symptoms was not feasible. To reduce variability in severity and acuity of LBP, we attempted to mitigate pre-existing back pain by defining a clean period, excluding individuals with health conditions that cause LBP symptoms for 6 months prior to index date, which is consistent with other studies [10, 13]. Additionally, the Other category included radiology, anesthesiology, and neurosurgery. As these providers are not typical first providers seen for LBP, further investigation may be warranted to understand why these providers were seen first. This finding may suggest that other providers were seen prior to the clean period. Related, a high percentage of individuals who saw EM physicians received long-term opioids despite these physicians not seeing individuals longitudinally after the first visit. Hence, additional work is needed to determine how previous LBP experiences beyond the 6-month period along with factors like provider education and experience with LBP affects provider cost and utilization.

Despite using a robust econometric approach to establish causality between first provider seen and health outcomes, finding a suitable instrument that meets the necessary requirements and has face validity remains a challenge. While both copay and differential distance have support in the literature and pass tests of independence and strength, both instruments may have influence on not only the first visit but also downstream visits. For example, the choice of the initial provider based on distance translates to time costs and may affect decisions about choice of providers or decisions about follow up after the first visit. Similarly, patients may be influenced by copays that may be apparent with repeat visits to a provider. Also, although 2SRI is generally accepted as the appropriate method to assess nonlinear relationships between treatment and outcome, there is ongoing debate about how choice of residuals (e.g., raw, deviance, Anscombe), rarity of outcomes, and setting may affect bias in estimates [33].

## Discussion

This national study demonstrates that health care utilization, cost, and opioid use significantly varied based on the first health care provider seen by individuals with LBP. Our study finds that the results from smaller-scale studies based on single regions or insurance providers persist when leveraging a large sample and using robust methods to adjust for selection bias; substantially different results would have been otherwise concerning given the consistency of results across studies [12, 13]. In other words, our study gives confidence that care beginning with more conservative providers (e.g., PT, Chiro, and Acu) may in fact significantly lower use of potentially unnecessary and costly imaging services and prescription opioids. While we found copay for the initial provider visit to be a valid instrument, producing similar results as differential distance as an instrument, there are limitations in its face validity and in validating the strength of the instrument when using large datasets like national claims data. Additional studies using different datasets or considering different conditions to compare copay relative to differential distance as an instrument for the choice of provider is warranted.

The findings of this study were similar to other studies exploring the rate by provider type that acted as the entry point into the health care system for LBP. In a retrospective review of claims data in a southwestern University-based health care plan, Fritz and colleagues [10] reported primary care, chiropractic and physiatry providers were the three most common entry points into the health care system for LBP. Using a national sample, we found PCP, Chiro, and Ortho were seen most commonly as the entry point to health care. We assume that among the variables affecting choice of provider for entry point include state regulation, insurance policy, regional supply of providers, and personal beliefs and norms. Further study is required to determine the relative impact of these factors on provider type seeking behaviors.

The first health care provider seen is the entry point into the health care system and affects assessment and intervention strategies that lead to significant variance in health care utilization and cost. For example, expensive MRIs have been shown to be used more by Ortho and PM&R whereas individuals that first saw Chiro and Acu had significantly less MRI use over the one-year follow-up. This finding may be partially explained by legal and regulatory restrictions in ordering MRI and radiology based on provider type (e.g., Acu cannot order MRIs in the US). However, the restrictions do not explain the variance among approved prescribers (e.g., various physician types, PRNs). Dietrich et al. [35] found significant differences among US Military Health Services physician assistants, APRNs, and physicians in ordering MRI and radiography, opioids, and anti-inflammatory medications in individuals with LBP. Additionally, Cherkin, et al. [36] found that diagnostic test ordered for individuals with LBP varied by physician specialty rather than patient symptoms and findings and in some cases, diagnostic test was not aligned with current clinical practice guidelines. These studies suggest that differences among allowed prescribers may reflect practice habits or professional education differences. Further study is warranted.

The variances seen in early- and long-term opioid prescriptions rates are concerning given the ongoing opioid crisis. The study demonstrated that EM, PM&R, and APRN had higher early and long-term opioids prescription rates than for Acu, Chiros, or PTs. We may assume since Acu, Chiros, and PTs cannot currently prescribe opioids, they will have lower prescription rates. However, our study investigated the full episode of care with 1 year follow-up including all subsequent health care utilization. It was likely that individuals initiating care with Acu, Chiros, or PTs saw allowable prescribers before or after the initial visit. Our study suggests that the initial provider may play an important role in setting the course of treatment and providers seen throughout the full episode of care. Evidence suggests that health care provider habits may influence patient treatment. Barnett, et al. [37] report that individuals visiting the ED and treated by high intensity opioid prescribers were more likely to be long-term opioid users. Additionally, the opioid prescription rate, although relatively low for some providers, remains a concern and is incongruent with most clinical practice guidelines [38].

A surprising finding was the high rate of ED visits for individuals with LBP seeing Chiros first. In a secondary analysis of 2012 National Health Interview Survey data, Forte and Maiers [39] found one in four individuals 65 years or older who saw chiropractors reported at least one ED visit in the prior 12 months. Interestingly, the ED visit rate between those that reported having manipulation was comparable to controls (no manipulation). Further research may prove helpful in understanding these findings.

Our findings show that POS plans were more often held by individuals who seek more conservative or complimentary providers, perhaps suggesting opportunities for modifications to HMO and PPO plan provisions. Notably, the presence of high deductible plans was equally common for individuals across all providers. Given the need to tighten economic spending due to COVID, US policymakers are seeking ways to encourage individuals to get low cost but high value care (i.e., care that follows clinical practice guidelines). While we have limited information on health outcomes, our findings support that some conservative providers deliver lower cost while providing care aligned with clinical practice guidelines. With scope of practice laws being relaxed across states temporarily due to the COVID crisis, the country is going through a natural experiment as individuals have greater access to varied providers. While these practice laws may change, our results suggest that policymakers should consider less restrictive laws so as to not limit access to effective providers.

## Conclusions

This study found that health care utilization and cost varied by the health care provider type seen on the initial visit for individuals with LBP. The first health care provider seen may also affect the use of evidence-based clinical practice guidelines. Finally, early and long-term opioid use for individuals with LBP varied significantly based on the initial health care provider. While a prospective randomized control trial remains the gold standard for controlling for selection bias, this study provides a large-scale, national view of the complex and real-world relationship between the first provider and subsequent health care utilization and costs. While continued research is needed to fully understand the reasons for cost and utilization differences among the providers, this study suggests that US policymakers should consider current insurance, regulatory and government policy to encourage individuals to seek care from providers that follow clinical practice guidelines.

## Supplementary Information

**Additional file 1.**

**Additional file 2.**

## Data Availability

The data that support the findings of this study are available from Health Care Cost Institute (HCCI, https://healthcostinstitute.org) but restrictions apply to the availability of these data, which were used under license for the current study, and so are not publicly available. HCCI is an independent, non-profit organization that licenses access to insurance claims data for a fee via secure enclave. Data are however available from the authors upon reasonable request and with permission of HCCI that may require an additional fee.
