# Supplementary material for: Where to start? A two stage residual inclusion approach to estimating influence of the initial provider on health care utilization and costs for low back pain in the US
Source: BMC Health Serv Res. 2022 May 23;22:694. doi: 10.1186/s12913-022-08092-1 (PMC9128255; doi:10.1186/s12913-022-08092-1)
Supplement: Supplementary file 1 — Additional file 1. [file 12913_2022_8092_MOESM1_ESM.docx]

**Supplement 1**

Table A: Exclusion Criteria

| **Description** | **ICD 9 Codes** | **ICD 10 codes** | **CPT Codes** |
| --- | --- | --- | --- |
| **Non MSK reasons for LBP** |  |  |  |
| Calculus of kidney | 592.xx | N20.0, N20.1, N20.9 |  |
| Calculus of gallbladder without mention of cholecystitis | 574.2 | K80.20 |  |
| Polynephritis | 590.9 | N15.9 |  |
| UTI (site not specified) | 599 | N39.0 |  |
| Urinary tract infection | V13.02 | Z87.440 |  |
| Neoplasms | 140.xx-239.xx | C00.xx - D49.xx |  |
| Osteoporosis | V17.81, V82.81 | Z82.62, Z13.820 |  |
| cauda equina syndrome | 344.6 | G83.4 |  |
| Osteomyelitis, periostitis and other infections involving bone | 730.xx | M86.xx, M89.60, M89.619, M89.629, M89.639, M89.649, M89.659, M89.669, M89.679, M89.968, M89.69, M90.80, M90.810, M90.829, M90.830, M90.849, M90.859, M90.869, M90.879, M90.88, M90.89, M46.20, M46.30 |  |
| major osseous deficit | 731.3 | M89.70 |  |
| fracture of spine or pelvis | 805.x - 809.x, 733.13-733.15 or 733.96-733.98 | S12.9XXA, S12.000A, S12.001A, S12.100A, S12.101A, S12.200A, S12.201A, S12.300A, S12.301A, S14.101A, S14.102A, S14.103A, S14.104A, S22.0XXX, S32.501A, S32.502A, S32.509A, S32.501B, S32.502B, S32.509B, S32.9XXA, S32.9XXB, S22.99XA, S22.9XXB, S32.009A, S32.019A, S32.029A, S32.039A, S32.049A,S32.059A, S34.109A, S34.119A, S34.129A, S34.101A, S34.111A,S34.121A, S34.102A, S34.112A, S34.122A,S34.103A,S34.113A, S34.123A,S34.104A, S34.114A, S34.124A, S34.105A, S34.115A, S34.125A, M84.453A, M84.750A, M84.359A, M84.353A, M84.350A, M48.50XA, M80.08XA, S32.1 |  |
| Intraspinal abscess | 324.1 | G06.1 |  |
| **Other Exclusions** |  |  |  |
| low back surgery |  |  | 00630, 00670, 20930, 20936, 22102, 22103, 22224, 22226, 22558, 22585, 22612,22630, 22802, 22840, 22842–22844, 22851, 62287, 63005, 63011, 63012, 63030, 63035, 63042, 63047, 63048, 63088–63091,63185, 63190, 63200, 63267, 63272, 63290, 63303, 63047, 63048, 64622, 64623 |
| Opioid dependence |  | F11.20 |  |
| substance abuse disorder |  | 291.x, 303.x, 304.x, 305, 305.2x - 305.9x, 648.3 |  |
| opioid use disorder | 304.00' 305.5 |  |  |
| proxy for opioid use disorder |  |  | 99233, 99221, 99222, 99231, 99232, 90805, 90807, 90809, 90801, 90804, 90806, 90808, 90853, 90899 |

Table B Diagnostic Inclusion Criteria

| **Descriptor** | **ICD 9** | **ICD 10** | **Descriptor2** | **ICD 93** | **ICD 104** |
| --- | --- | --- | --- | --- | --- |
| Sacroiliitis, not elsewhere classified | 720.2 | M46.1 | disorders of the sacrum | 724.6 | M53.3 |
| lumbosacaral spondylosis without melopahty | 721.3 | M47.817 | other unspecified back disorders | 724.9 | M53.9 |
| Spondylosis with myelopathy, lumbar region | 721.42 | M47.16 | Acquired spondylolisthesis | 738.4 | M43.00, M43.01 |
| lumbar disc displacement | 722.1 | M51.26, M51.27 | Nonallopathic lesions, lumbar region | 739.3 | m99.03 |
| Displacement of intervertebral disc, site unspecified, without myelopathy | 722.2 | M51.9 | Nonallopathic lesions, sacral region | 739.4 | M99.04 |
| Degeneration of thoracic or thoracolumbar intervertebral disc | 722.51 | M51.35 | Spondylolysis, lumbosacral region | 756.11 | Q76.2 |
| lumbar/lumbosacral disc displacement | 722.52 | M51.36, M51.37 | spondylolisthesis | 756.12 | Q76.2 |
| lumbar disc disease with myelopathy | 722.73 | M51.06, M51.07 | sprain-lumbosacral region | 846.x | S33.8XXA |
| other disc disorder - lumbar region | 722.93 | M46.47, M51.86, M51.87 | sprain -sacroiliac | 846.1 | S33.6XXA |
| spinal stenosis - lumbar | 724.02 | M48.06 | sprain- other specified sites of sacroiliac region | 846.8 | S33.8XXA |
| lumbago | 724.2 | M54.5 | sprain- unspecified sites of sacroiliac region | 846.9 | S33.9XXA |
| sciatica | 724.3 | M54.30 | sprain-lumbar region | 847.2 | S33.5XXA |
| thoracic or lumbosacral neuritis or radiculitis, unspecified | 724.4 | M54.214-M54.17 | sprain - sacrum | 847.3 | S33.8XXA |
| backache, unspecified | 724.5 | M54.89, M54.9 |  |  |  |
