# Supplementary material for: Where to start? A two stage residual inclusion approach to estimating influence of the initial provider on health care utilization and costs for low back pain in the US
Source: BMC Health Serv Res. 2022 May 23;22:694. doi: 10.1186/s12913-022-08092-1 (PMC9128255; doi:10.1186/s12913-022-08092-1)
Supplement: Supplementary file 2 — Additional file 2. [file 12913_2022_8092_MOESM2_ESM.docx]

**Supplement 2**

Differential distance was defined as the difference of: 1) the distance between a patient and the first provider of choice, and, 2) the distance between a patient and the closest alternative provider. The extra distance that a patient may go to see their provider of choice over a local alternative captures the otherwise unobserved set of factors that goes into a patients’ choice of first provider. Specifically, differential distance was defined as the difference between: 1) the shortest distance on a sphere (i.e., great-circle distance using the Haversine formula provided by the National Bureau of Economic Research)(1) between the centroid of the zip code associated with the individual and the centroid of the zip code associated with the first provider seen, and 2) the shortest distance between the centroid of the individual zip code and any other provider not seen by the individual on the index date but is within the individual’s plan. Distances were limited to 100 miles due to computational limitations. We also tested road mile and drive time distances as alternatives obtained from zip code pairs from the Medicare beneficiary file and calculated using a SAS program (2) which was found to be a strong instrument in a related study. (3)

In testing the difference between copay or differential distance as the instrument variable, we found the parameter estimates were nearly identical with slightly more narrow standard errors at the fifth or sixth decimal place using copay. The tradeoff was the loss of information from the nearly three-quarter of a million observations missing distance information. Using deviance residuals in place of raw residuals also produced nearly identical results. Adding state dummies produced similar consistent parameter estimates but we did not include them at risk of overfitting.

While causality cannot be established in the first stage, higher copay was slightly positively associated with the probability of seeing an APRN, Ortho, PM&R or EM first, and negatively associated with the probability of seeing a PCP, Chiro, PT or Acu. Using differential distance, individuals were more likely to travel further to see EM, PM&R, or Ortho first compared to an alternate provider, while individuals were less likely to travel further to see a Chiro, PT or PCP (distance appeared to have little impact on choice of seeing an APRN or Acu).

References

(1) The National Bureau of Economic Research. Zip code distance database. 2017; Available at: <https://data.nber.org/data/zip-code-distance-database.html>. Accessed February 4, 2021.

(2) SAS Communities Library. Driving distances and drive times using SAS and google maps. 2018; Available at: <https://communities.sas.com/t5/SAS-Communities-Library/Driving-Distances-and-Drive-Times-using-SAS-and-Google-Maps/ta-p/475839>. Accessed February 5, 2021.

(3) Andrilla CHA, Coulthard C, Patterson DG. Prescribing Practices of Rural Physicians Waivered to Prescribe Buprenorphine. Am J Prev Med 2018 June 01;54(6 Suppl 3):S208-S214.

stylefix

**Table A: First stage of 2SRI predicting first provider seen using copay as the instrument**

| **Reference=PT** | **Chiro** | | **Acu** | | **APRN** | | **PCP** | | **PM&R** | | **Ortho** | | **EM** | | **Other** | |
| --- | --- | --- | --- | --- | --- | --- | --- | --- | --- | --- | --- | --- | --- | --- | --- | --- |
|  | **Coeff** | **SE** | **Coeff** | **SE** | **Coeff** | **SE** | **Coeff** | **SE** | **Coeff** | **SE** | **Coeff** | **SE** | **Coeff** | **SE** | **Coeff** | **SE** |
| **Copay** | 0.003 | 0.000 | -0.015 | 0.000 | 0.011 | 0.000 | 0.006 | 0.000 | 0.017 | 0.000 | 0.019 | 0.000 | 0.014 | 0.000 | 0.013 | 0.000 |
| **Female** | -0.276 | 0.007 | 0.177 | 0.012 | -0.073 | 0.010 | -0.276 | 0.007 | -0.117 | 0.008 | -0.206 | 0.008 | -0.258 | 0.009 | -0.126 | 0.007 |
| **Age 18-34 (reference)** |  |  |  |  |  |  |  |  |  |  |  |  |  |  |  |  |
| **Age 35-44** | -0.071 | 0.011 | 0.350 | 0.018 | 0.038 | 0.017 | 0.140 | 0.011 | 0.331 | 0.015 | 0.111 | 0.014 | -0.199 | 0.014 | 0.066 | 0.011 |
| **Age 45-54** | -0.163 | 0.010 | 0.057 | 0.018 | 0.069 | 0.016 | 0.222 | 0.010 | 0.573 | 0.014 | 0.377 | 0.013 | -0.412 | 0.014 | 0.230 | 0.010 |
| **Age 55-64** | -0.363 | 0.010 | -0.413 | 0.020 | -0.046 | 0.016 | 0.169 | 0.010 | 0.633 | 0.014 | 0.514 | 0.013 | -0.775 | 0.014 | 0.262 | 0.010 |
| **Age 65-74** | -0.472 | 0.013 | -0.734 | 0.030 | -0.548 | 0.023 | -0.272 | 0.013 | 0.220 | 0.017 | 0.461 | 0.016 | -1.262 | 0.020 | -0.115 | 0.013 |
| **Age 75+** | -0.620 | 0.016 | -1.105 | 0.049 | -0.454 | 0.026 | -0.229 | 0.016 | 0.219 | 0.020 | 0.543 | 0.019 | -1.141 | 0.024 | 0.039 | 0.016 |
| **Medicare Advantage Plan** | 0.256 | 0.012 | -1.142 | 0.041 | 0.441 | 0.021 | 0.778 | 0.012 | 1.093 | 0.015 | 0.561 | 0.014 | 0.607 | 0.018 | 0.816 | 0.012 |
| **Elixhauser Index (#)** | -0.023 | 0.022 | -0.715 | 0.060 | 0.731 | 0.028 | 0.834 | 0.021 | 0.640 | 0.024 | 0.174 | 0.025 | 0.486 | 0.028 | 1.052 | 0.021 |
| **PPO/EPO (reference)** |  |  |  |  |  |  |  |  |  |  |  |  |  |  |  |  |
| **POS** | 0.149 | 0.009 | 0.249 | 0.017 | 0.394 | 0.015 | -0.010 | 0.009 | -0.052 | 0.011 | -0.024 | 0.010 | -0.025 | 0.012 | -0.179 | 0.009 |
| **HMO** | -0.241 | 0.013 | -0.879 | 0.037 | 0.469 | 0.019 | 0.544 | 0.012 | 0.470 | 0.014 | 0.308 | 0.014 | 0.730 | 0.016 | 0.413 | 0.012 |
| **Other** | 0.033 | 0.019 | -1.218 | 0.075 | 0.484 | 0.030 | 0.133 | 0.019 | 0.287 | 0.024 | 0.231 | 0.022 | 0.013 | 0.033 | 0.280 | 0.019 |
| **Not in CDHP (reference)** |  |  |  |  |  |  |  |  |  |  |  |  |  |  |  |  |
| **High Deductible Plan** | 0.170 | 0.008 | -0.305 | 0.015 | 0.146 | 0.013 | 0.078 | 0.008 | 0.254 | 0.011 | 0.366 | 0.010 | 0.246 | 0.011 | 0.131 | 0.008 |
| **Unknown High Deductible** | -0.233 | 0.015 | -0.282 | 0.044 | 0.208 | 0.022 | -0.570 | 0.015 | -0.810 | 0.018 | -0.579 | 0.017 | -0.901 | 0.026 | -0.545 | 0.014 |
| **County has Low Education** | -0.045 | 0.015 | 0.843 | 0.023 | -0.024 | 0.021 | 0.017 | 0.014 | -0.407 | 0.018 | -0.055 | 0.016 | -0.060 | 0.019 | -0.116 | 0.014 |
| **County has Low Employ** | -0.044 | 0.014 | -0.920 | 0.037 | 0.378 | 0.020 | 0.317 | 0.014 | 0.208 | 0.017 | 0.193 | 0.016 | 0.214 | 0.019 | 0.219 | 0.014 |
| **County Uninsured %** | 0.019 | 0.001 | -0.027 | 0.002 | 0.033 | 0.001 | 0.060 | 0.001 | 0.046 | 0.001 | 0.065 | 0.001 | 0.063 | 0.001 | 0.067 | 0.001 |
| **Metropolitan (reference)** |  |  |  |  |  |  |  |  |  |  |  |  |  |  |  |  |
| **Micropolitan (UIC)** | 0.838 | 0.017 | -1.506 | 0.065 | 1.200 | 0.021 | 0.506 | 0.017 | 0.100 | 0.020 | 0.136 | 0.019 | 0.187 | 0.023 | 0.442 | 0.017 |
| **Noncore (UIC)** | 0.631 | 0.016 | -0.541 | 0.044 | 0.788 | 0.023 | 0.218 | 0.016 | -0.172 | 0.020 | -0.085 | 0.019 | -0.104 | 0.024 | 0.213 | 0.016 |
| **Unrestricted (reference)** |  |  |  |  |  |  |  |  |  |  |  |  |  |  |  |  |
| **PT Provisions** | -0.041 | 0.009 | 0.487 | 0.017 | -0.206 | 0.014 | 0.008 | 0.009 | 0.121 | 0.011 | 0.325 | 0.011 | 0.037 | 0.013 | -0.070 | 0.008 |
| **PT Limited** | 0.403 | 0.011 | -0.406 | 0.026 | 0.181 | 0.017 | 0.441 | 0.011 | 0.506 | 0.014 | 0.805 | 0.014 | 0.338 | 0.016 | 0.322 | 0.011 |
| **Constant** | 2.027 | 0.015 | -0.944 | 0.029 | -1.474 | 0.024 | 1.171 | 0.015 | -1.129 | 0.019 | -1.221 | 0.018 | -0.694 | 0.020 | 1.280 | 0.015 |

**Table B: First stage of 2SRI predicting first provider seen using differential distance as the instrument**

| **Reference = PT** | **Chiro** | | **Acu** | | **APRN** | | **PCP** | | **PM&R** | | **Ortho** | | **EM** | | **Other** | |
| --- | --- | --- | --- | --- | --- | --- | --- | --- | --- | --- | --- | --- | --- | --- | --- | --- |
|  | **Coeff** | **SE** | **Coeff** | **SE** | **Coeff** | **SE** | **Coeff** | **SE** | **Coeff** | **SE** | **Coeff** | **SE** | **Coeff** | **SE** | **Coeff** | **SE** |
| **Diff Distance** | -0.003 | 0.000 | 0.016 | 0.000 | 0.010 | 0.000 | -0.001 | 0.000 | 0.024 | 0.000 | 0.021 | 0.000 | 0.020 | 0.000 | 0.024 | 0.000 |
| **Female** | -0.276 | 0.007 | 0.185 | 0.013 | -0.083 | 0.012 | -0.284 | 0.007 | -0.129 | 0.009 | -0.220 | 0.008 | -0.279 | 0.010 | -0.124 | 0.007 |
| **Age 18-34 (reference)** |  |  |  |  |  |  |  |  |  |  |  |  |  |  |  |  |
| **Age 35-44** | -0.089 | 0.011 | 0.334 | 0.018 | 0.026 | 0.019 | 0.112 | 0.012 | 0.326 | 0.016 | 0.105 | 0.015 | -0.192 | 0.015 | 0.066 | 0.012 |
| **Age 45-54** | -0.182 | 0.011 | 0.046 | 0.019 | 0.048 | 0.018 | 0.192 | 0.011 | 0.572 | 0.015 | 0.369 | 0.014 | -0.406 | 0.015 | 0.235 | 0.011 |
| **Age 55-64** | -0.396 | 0.011 | -0.414 | 0.021 | -0.070 | 0.018 | 0.129 | 0.011 | 0.627 | 0.015 | 0.487 | 0.014 | -0.787 | 0.016 | 0.269 | 0.011 |
| **Age 65-74** | -0.556 | 0.014 | -0.693 | 0.032 | -0.627 | 0.026 | -0.322 | 0.014 | 0.183 | 0.019 | 0.373 | 0.017 | -1.323 | 0.024 | -0.090 | 0.014 |
| **Age 75+** | -0.727 | 0.017 | -1.015 | 0.053 | -0.551 | 0.030 | -0.278 | 0.017 | 0.176 | 0.022 | 0.458 | 0.021 | -1.192 | 0.029 | 0.066 | 0.017 |
| **Medicare Advantage Plan** | 0.309 | 0.014 | -1.143 | 0.045 | 0.536 | 0.024 | 0.735 | 0.014 | 1.168 | 0.017 | 0.651 | 0.016 | 0.370 | 0.022 | 0.774 | 0.014 |
| **Elixhauser Index (#)** | -0.001 | 0.024 | -0.717 | 0.064 | 0.720 | 0.032 | 0.825 | 0.023 | 0.650 | 0.026 | 0.153 | 0.027 | 0.360 | 0.033 | 0.987 | 0.023 |
| **PPO/EPO (reference)** |  |  |  |  |  |  |  |  |  |  |  |  |  |  |  |  |
| **POS** | 0.126 | 0.009 | 0.213 | 0.018 | 0.362 | 0.016 | 0.027 | 0.009 | 0.030 | 0.012 | 0.048 | 0.011 | 0.062 | 0.014 | -0.088 | 0.009 |
| **HMO** | -0.187 | 0.014 | -0.942 | 0.039 | 0.464 | 0.022 | 0.581 | 0.013 | 0.554 | 0.015 | 0.412 | 0.015 | 0.765 | 0.019 | 0.510 | 0.013 |
| **Other** | 0.019 | 0.021 | -1.173 | 0.082 | 0.434 | 0.035 | 0.078 | 0.021 | 0.199 | 0.027 | 0.099 | 0.025 | -0.125 | 0.042 | 0.131 | 0.021 |
| **Not in CDHP (reference)** |  |  |  |  |  |  |  |  |  |  |  |  |  |  |  |  |
| **High Deductible Plan** | 0.133 | 0.008 | -0.136 | 0.015 | -0.002 | 0.014 | 0.002 | 0.009 | 0.004 | 0.011 | 0.088 | 0.010 | 0.039 | 0.012 | -0.052 | 0.008 |
| **Unknown High Deductible** | -0.283 | 0.016 | -0.266 | 0.047 | 0.216 | 0.025 | -0.485 | 0.016 | -0.767 | 0.019 | -0.559 | 0.019 | -0.637 | 0.029 | -0.451 | 0.015 |
| **County has Low Education** | -0.066 | 0.016 | 0.875 | 0.024 | -0.197 | 0.024 | -0.106 | 0.016 | -0.512 | 0.020 | -0.120 | 0.018 | -0.091 | 0.022 | -0.221 | 0.016 |
| **County has Low Employ** | -0.027 | 0.017 | -1.465 | 0.059 | 0.506 | 0.024 | 0.370 | 0.017 | 0.225 | 0.021 | 0.151 | 0.020 | 0.218 | 0.025 | 0.260 | 0.017 |
| **County Uninsured %** | 0.023 | 0.001 | -0.028 | 0.002 | 0.048 | 0.001 | 0.073 | 0.001 | 0.053 | 0.001 | 0.074 | 0.001 | 0.071 | 0.001 | 0.077 | 0.001 |
| **Metropolitan (reference)** |  |  |  |  |  |  |  |  |  |  |  |  |  |  |  |  |
| **Micropolitan (UIC)** | 0.606 | 0.018 | -1.748 | 0.079 | 0.972 | 0.024 | 0.244 | 0.018 | -0.145 | 0.023 | -0.140 | 0.022 | -0.126 | 0.029 | 0.141 | 0.018 |
| **Noncore (UIC)** | 0.307 | 0.023 | -1.600 | 0.105 | 0.704 | 0.031 | 0.021 | 0.023 | -0.424 | 0.030 | -0.425 | 0.029 | -0.463 | 0.038 | -0.015 | 0.023 |
| **PT Provisions** | -0.032 | 0.009 | 0.572 | 0.019 | -0.326 | 0.015 | -0.061 | 0.009 | 0.140 | 0.012 | 0.397 | 0.013 | 0.042 | 0.015 | -0.088 | 0.009 |
| **PT Limited** | 0.438 | 0.012 | -0.342 | 0.028 | 0.038 | 0.019 | 0.354 | 0.012 | 0.492 | 0.016 | 0.865 | 0.015 | 0.283 | 0.018 | 0.257 | 0.012 |
| **Constant** | 2.047 | 0.016 | -1.253 | 0.032 | -1.430 | 0.026 | 1.168 | 0.016 | -1.179 | 0.021 | -1.252 | 0.020 | -0.839 | 0.023 | 1.038 | 0.016 |

**Select results using differential distance as the instrumental variable:**

Figure A. Health Care Utilization Adjusted Rates by First Provider Seen for Low Back Pain (using differential distance)

Table C. Marginal Effects of Patient Utilizing Health Care Services and Experiencing Serious Illnesses in 12-Months after First Provider Seen for Low Back Pain (using differential distance)

|  | **Early Opioid Rx** | | **Long Opioid Rx** | | **Had MRI/CT** | | **Had Radiography** | | **Had ED Visit** | | **Had Hospitalization** | | **Had Surgery** | | **Had Serious Illness** | |
| --- | --- | --- | --- | --- | --- | --- | --- | --- | --- | --- | --- | --- | --- | --- | --- | --- |
|  | **Margin** | **SE** | **Margin** | **SE** | **Margin** | **SE** | **Margin** | **SE** | **Margin** | **SE** | **Margin** | **SE** | **Margin** | **SE** | **Margin** | **SE** |
| PT | 3.1 | 0.060 | 1.4 | 0.041 | 16.1 | 0.119 | 11.1 | 0.102 | 16.0 | 0.119 | 7.2 | 0.084 | 2.3 | 0.049 | 22.8 | 0.133 |
| Chiro | 1.6 | 0.016 | 0.5 | 0.010 | 6.7 | 0.029 | 17.5 | 0.044 | 42.6 | 0.058 | 5.7 | 0.028 | 0.7 | 0.010 | 19.4 | 0.047 |
| Acu | 1.2 | 0.069 | 0.4 | 0.046 | 5.7 | 0.131 | 5.9 | 0.131 | 17.9 | 0.212 | 8.2 | 0.168 | 0.5 | 0.041 | 21.8 | 0.232 |
| APRN | 10.8 | 0.143 | 5.0 | 0.100 | 17.9 | 0.179 | 19.6 | 0.186 | 19.0 | 0.181 | 7.6 | 0.124 | 2.6 | 0.074 | 21.4 | 0.189 |
| PCP | 9.3 | 0.033 | 3.3 | 0.020 | 16.2 | 0.042 | 17.5 | 0.044 | 16.4 | 0.042 | 6.3 | 0.028 | 1.9 | 0.016 | 19.8 | 0.045 |
| PM&R | 11.0 | 0.082 | 6.1 | 0.060 | 27.9 | 0.124 | 20.4 | 0.112 | 19.3 | 0.108 | 8.4 | 0.073 | 3.6 | 0.051 | 24.4 | 0.114 |
| Ortho | 7.7 | 0.066 | 2.3 | 0.037 | 36.5 | 0.122 | 46.7 | 0.128 | 17.4 | 0.096 | 9.6 | 0.072 | 6.5 | 0.062 | 25.5 | 0.107 |
| EM | 12.0 | 0.128 | 1.7 | 0.055 | 15.8 | 0.146 | 19.7 | 0.155 | 31.3 | 0.178 | 7.8 | 0.110 | 1.9 | 0.056 | 21.0 | 0.164 |

Figure B: Early and Long Opioid Prescription (Adjusted Rates) by First Provider Seen for Low Back Pain (using differential distance)

Table D. Healthcare Category Ranking by First Provider Seen (Highest Use=1, Lowest Use = 8) (using differential distance)

|  | PT | Chiro | Acu | APRN | PCP | PM&R | Ortho | EM |
| --- | --- | --- | --- | --- | --- | --- | --- | --- |
| Early Opioid Rx | 6 | 7 | 8 | 3 | 4 | 2 | 5 | 1 |
| Long Opioid Rx | 6 | 7 | 8 | 2 | 3 | 1 | 4 | 5 |
| MRI/CT | 5 | 7 | 8 | 3 | 4 | 2 | 1 | 6 |
| Any Radiography | 7 | 5* | 8 | 4 | 5* | 2 | 1 | 3 |
| Had ED Visit | 8 | 1 | 5 | 4 | 7 | 3 | 6 | 2 |
| Hospitalization | 6 | 8 | 3 | 5 | 7 | 2 | 1 | 4 |
| Had Surgery | 4 | 7 | 8 | 3 | 5* | 2 | 1 | 5* |
| Had Serious Illness | 3 | 8 | 4 | 5 | 7 | 2 | 1 | 6 |
